# Practice behaviors as trigger factor for the onset of Musicians’ Dystonia

**DOI:** 10.1007/s00702-023-02689-4

**Published:** 2023-08-26

**Authors:** Edoardo Passarotto, Johanna Doll-Lee, Eckart Altenmüller, André Lee

**Affiliations:** 1 Institute of Music Physiology and Musicians’ Medicine, University of Music, Drama and Media Hannover, Hannover, Germany; 2 Department of Neurology, Hannover Medical School, Hannover, Germany; 3 Department of Neurology, Klinikum Rechts der Isar, Technical University of Munich, Munich, Germany

**Keywords:** Musicians’ Dystonia, Practice behaviors, Practice quantity, Trigger factor

## Abstract

Musician’s Dystonia (MD) is a task-specific movement disorder that results in an involuntary cramping of muscles involved in playing an instrument such as the upper limbs or the embouchure. It is usually painless and occurs in general only at the instrument. The pathophysiology of MD is not completely understood. The present study aimed at assessing differences in practice behaviors between pianists affected by MD and Healthy Controls (HC) in the years preceding the onset of the disease. Thus, we investigated to what extent *practice quantity* can be considered a trigger of Musicians’ Dystonia. The results showed that despite comparable practice behaviors in childhood, MD pianists incremented the amount of daily practice to a greater extent than their healthy colleagues, especially in the second and in the third decade of life. Thus, subsequent logistic regression analysis showed that high amounts of daily practice might significantly increase the risk of developing MD. Furthermore, gender-related differences in practice behaviors across groups were identified, indicating that male pianists from the MD group might not have practiced significantly more than HC male pianists before the onset of the disease. To the authors’ knowledge, these are the first empirical evidence of the role of dysfunctional practice behaviors in triggering MD, which has clinical and educational implications.

## Introduction

Musician’s Dystonia (MD) is a task-specific movement disorder. It results in involuntary cramping of muscles involved in playing an instrument such as the hand or embouchure, thus bringing about a loss of dexterity of highly trained fine motor movements (Furuya et al. 2015). It is usually painless and occurs in general only at the instrument. The pathophysiology of MD is not completely understood, but several risk factors are known, among them genetic predisposition, psychological traits, traumas, and playing-related injuries (Alpheis et al. 2022; Charness et al. 1996; Defazio et al. 1998; Doll-Lee et al. 2023; Schmidt et al. 2013). Another essential factor seems to be practice burden, i.e., highly repetitive movements performed over a long period of time (Altenmüller 2003; Lederman 1991). This is corroborated by an influential animal model with primates, where it was shown that repetitive movements performed over a long period of time could induce a dystonic muscle contractions in the hand (Byl et al. 1996). In musicians, repetitive movements over a long period of time occur during practice periods to consolidate highly controlled fine motor sequences. The practice burden typically varies among professional musicians depending upon the instrument. For example, probably due to the highly virtuosic demands of their respective literature and the high competitiveness in their field, professional violinists as well as pianists typically report a particularly high amount of daily practice (Hallam et al. 2020). The same applies to guitarists, which is reflected in the frequency of occurrence of MD in those instruments compared to others (Lim and Altenmüller 2003a). The role of high-precision motor movements is further highlighted by the notion that MD was first documented in the rising of instrumental virtuosity in the nineteenth century with Robert Schumann being the first to report a loss of control in highly trained fine motor patterns (Altenmüller 2005).

Identifying risk factors is of foremost relevance as there is still no causal therapy available for the treatment of MD. Symptomatic therapies include anticholinergic drugs like Trihexyphenidyl, which is, however, not well tolerated by patients and shows only limited effects (Jabusch et al. 2005). In Musician’s Dystonia of the upper or lower limb, the injection of Botulinum toxin, which is a standard treatment for Dystonia (Dressler et al. 2021), can ameliorate the dystonic cramp to such an extent that often allows the patients to continue their professional career (Dressler et al. 2016; Lee et al. 2021). Given the complexity of embouchure dystonia, treatment options are unsatisfactory. For this reason, it is important for research to determine modifiable risk factors such as practice behaviors as a means of primary prevention.

Yet, to our knowledge, no study has been conducted so far to systematically compare practice behavior in MD patients compared to healthy controls while controlling for instrumental differences and profession. We therefore present a study of practice behavior in professional pianists with specialist-diagnosed MD compared to healthy professional pianists.

Based on the results of the above studies, which indicate an important influence of highly repetitive movements over an extended period of time, we hypothesized that the amount of daily practice time would differ between pianists with and without dystonia. In addition, because a striking gender disparity has been described in that significantly more men than women are affected by MD (Jabusch et al. 2005), we also hypothesized that practicing behavior would differ between male and female participants.

## Methods

### Design

The study investigated practice behaviors between 5 and 30 years of age in healthy pianists and pianists affected by MD. To ensure comparability, we included only professional pianists who at the time of filling in the questionnaire were either studying or had completed a degree in piano performance or piano teaching at a university of music. We included measures of practice quantity at different timeframes. Data from pianists affected by MD were collected using a questionnaire filled out by patients treated and diagnosed by a neurologist in an outpatient clinic specialized in musicians’ health. All participants gave informed consent.

The Central Ethics Committee at Leibniz University Hannover approved the present study.

### Participants

We included professional pianists above 30 years of age with a university degree in piano performance. Participants were divided into two groups: the MD group (*N* = 33), consisting of pianists affected by Focal Dystonia, and the Healthy Control group (HC group, *N* = 49), pianists who did not report any symptom comparable to MD during their entire career.

Only pianists who developed MD after the age of 30 were selected for the study: this allowed to investigate practice behaviors of interest (between 5 and 30 years) independently from the time course of the disease. Thus, the age at onset in our MD group, with a median of 40, was higher than the age at onset reported in other publications of about 34 (SD = 9.21), while the mean age when filling in the questionnaire was 55.47 years (SD = 9.37). 33.3% were females. The uneven distribution of gender is consistent with the literature on MD, reporting a higher incidence of the disease in male musicians (Alpheis et al. 2022; Altenmüller and Jabusch 2009; Lim and Altenmüller 2003b). HC pianists had a mean age of 41.44 years (SD = 11.84), and in their group, females and males were almost equally represented, constituting 53% and 47% of the sample, respectively. The difference in proportion of genders across groups was not significant (χ^2^ (1, *N* = 82) = 2.353, *p* = 0 0.125). Table 1 reports detailed descriptive statistics for both groups.

**Table 1:** Descriptive statistics for the MD and HC groups

	MD group (*N* = 33)	HC group (*N* = 49)
Age	55.47 (9.37)	41.44 (11.84)***
Gender		
Females	11	26
Males	22	23
Age at which participants started playing piano	7.25 (2.20)	6.65 (2.88)
Cumulative amount of practice at age 30	28,237 (9152)	26,455 (9483)
Average amount of daily practice between:		
5 and 10 years of age (tf1)	0.83 (0.47)	0.95 (0.75)
11 and 15 years of age (tf2)	1.83 (1.02)	2.13 (1.27)
16 and 20 years of age (tf3)	3.57 (1.70)	3.51 (1.65)
21 and 30 years of age (tf4)	4.69 (1.49)	4.01 (1.48)*
Median onset age of Musicians' Dystonia	40 (9.21)	–

*N* = 82

*tf* timeframe, *MD* Musicians’ Dystonia, *HC* healthy controls

*between groups *t* test significant at *p* < 0.05

***between groups *t* test significant at *p* < 0.001

### Materials

*Daily practice quantity* was estimated in terms of average amounts of practice per day over four *timeframes*: from 5 to 10 (*timeframe1*), from 11 to 15 (*timeframe2*), from 16 to 20 (*timeframe3*), and from 21 to 30 years of age (*timeframe4*). This information was used to compute *cumulative practice quantity*, which measures the cumulative number of hours practiced per day at different ages, namely at 10, 15, 20, and 30 years of age. Both *practice quantity* measures are reported in hours. In addition, participants provided information about the age at which they started practicing piano (*starting age*), their academic degrees in music, and their profession.

### Procedure

Participants were invited to fill out a short questionnaire online, investigating their musical background, the amount of practice achieved during their lifetime, previous playing-related injuries, as well as family health history.

### Data analyses

Bartlett tests were run to investigate whether the variances in *practice quantity* measures of the two groups were comparable: the results were non-significant (*p* > 0.05), indicating sufficient homogeneity of variances between samples. In the analyses, *group* was coded as HC group = 0 and MD group = 1, while *gender* as females = 0 and males = 1. Therefore, results take female pianists from the HC group as a reference.

Two Bayesian mixed-effects regression models were used to investigate the increment in the amount of *cumulative* and *daily practice quantity* over the years of interests. In the first regression model, *cumulative practice quantity* was entered as dependent variable and it was predicted by *age, gender, group,* and their two- and three-way interactions. The model also included random intercepts per *musician* with random slopes per *age* as random effects. For the analysis, a square-root transformation was applied to *cumulative practice quantity* to account for its right skewed distribution.

The second Bayesian mixed-effects model entered *daily practice quantity* as criterion, *timeframe, gender, group,* and their interactions as fixed effects as well as *musician* with random slopes per *timeframe* as random effects. *Timeframe* was treated as a four-level categorical variable, and the model intercept was set to 0*.*

Logistic regression models were run to assess the role of practice behaviors in triggering MD. Thus, group membership was predicted by the average amount of practice hours per day in the 21–30 years of age timeframe as well as *start_age, gender,* and the interaction practice_quality*gender. Random subsets of musicians from the HC group were selected for the analyses, to match the sample size of the MD group (*N* = 33). In the HC group, genders were equally represented, consisting of 17 male and 17 female pianists. A bootstrap technique was implemented. The sampling procedure and regression analyses were repeated for 1000 iterations. Appendix 3 shows the stability of regression coefficients and confidence intervals estimates at different bootstrap iterations: 1000 bootstrap iterations were deemed sufficient to ensure stable and reliable results.

## Results

### Cumulative practice quantity

The first part of the analyses aimed at identifying differences in *cumulative practice quantity* between HC and MD groups. *t* tests revealed no significant group differences in the amount of practice achieved at 30 years of age, *t*(80) = − 0.846, *p* = 0.399, nor in *starting age*, *t*(80) = − 1.019, *p* = 0.311. Subsequently, a Bayesian mixed-effect regression model was run to investigate the increment in *cumulative practice quantity* overtime. As shown in Table 2, meaningful positive age*group and age*gender interactions were identified, suggesting that male pianists and pianists from the MD group increased the amount of practice to a greater extent than their colleagues between 5 and 30 years of age. Nevertheless, the negative three-way age*group*gender interaction suggests possible compensations for the effect of *group* and *gender* over *age* in case of male pianists*,* with an estimated probability of approximately 83%.

**Table 2:** Group differences in cumulative practice quantity

	Estimate	Est. error	CI lower	CI upper
Fixed effects				
Intercept	− 21.06	4.51	− 30.02	− 12.28
Age	5.75	0.23	5.29	6.20
md_group	− 5.29	8.22	− 21.03	11.15
Male	− 13.26	6.64	− 26.17	− 0.16
age:md_group	0.89	0.44	0.04	1.74
age:male	0.77	0.34	0.09	1.44
md_group:male	− 5.56	10.70	− 26.16	14.95
age:md_group:male	− 0.80	0.57	− 1.94	0.32
Random effects				
Performer:				
sd(Intercept)	18.96	2.60	14.1	24.33
sd(age)	1.02	0.12	0.80	1.26
cor(Intercept, age)	− 0.45	0.13	− 0.67	− 0.16
sigma	9.12	0.55	8.12	10.30
Coefficients of determination				
Conditional R2	0.97	0.01	0.93	0.97
Marginal R2	0.81	0.01	0.79	0.82

The model takes female pianists from the HC group as a reference

*N* = 82; *CI* credible interval

### Daily practice quantity across timeframes

**Fig. 1:**
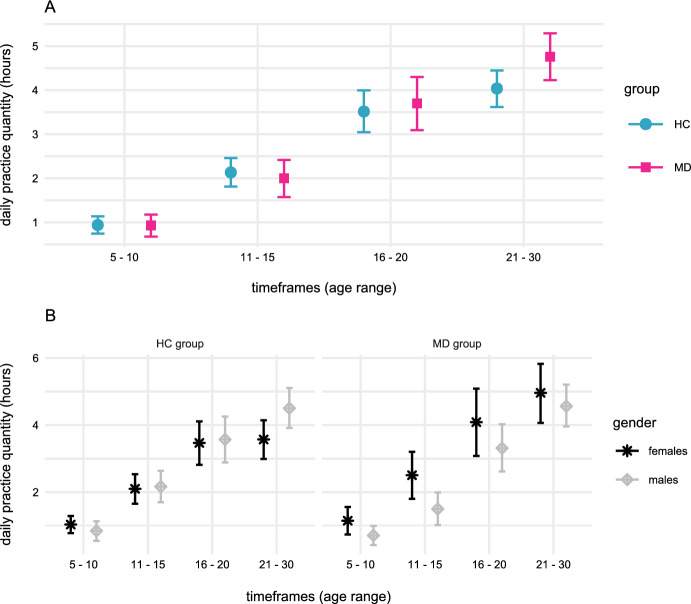
Daily practice quantity at different timeframes for HC and MD groups as well as *gender*. hours of *daily practice* at the respective timeframes for MD and HC groups (part A) as well as for *gender* for both groups (part B) were estimated from the Bayesian mixed-effects model coefficients reported in Appendix 1. For further information, see Sects. 2.3 Materials and 2.5 Data analyses

Subsequently, a Bayesian mixed-effects model with basis splines was used to assess group differences in *daily practice quantity* over the four timeframes considered in the study (see Sect.  2.3 Materials). As shown in Appendix 1 and Fig. 1, the results indicated meaningful interactions between *timeframe4 (i.e., between the age of 21 and 30)* and *group,* β = 1.27 [0.22, 2.34], as well as *timeframe4* and *gender,* β = 1.11 [0.28, 1.95]. Thus, male pianists and pianists from the MD group practiced more than their colleagues in *timeframe4*. As before, a negative three-way interaction between timeframe4*group*gender was identified, β = − 1.06 [− 2.45, 0.30], with a probability of approximately 88%.

Post hoc analyses of *daily practice quantity* confirmed that the MD group practiced more than HC group during *timeframe4*, *t* (68.47) = 2.06, *p* = 0.04. The same group-difference was identified between female MD and female HC pianists, *t* (29.11) = 3.10, *p* = 0.004. Nevertheless, these results did not generalize to male participants, as *daily practice quantity* during the same period was not significantly different between male pianists with and without MD, *t* (36.77) = 0.15, *p* = 0.875. For further information, see Appendix 2.

### Practice quantity as risk factor of Musicians’ Dystonia

Finally, a logistic regression model and a bootstrap procedure with 1000 iterations were run to quantify the contribution of *daily practice quantity* during the 21–30 years of age timeframe on the onset of MD (see Table 3). The results indicated a meaningful increase in the risk of getting MD due to *daily practice quantity*, β = 1.017 [0.127, 1.896], Odds Ratio = 2.765 [1.135, 6.656]. Once again, the negative two-way interaction between *daily practice quantity* and *gender* indicated a partial compensation of the effect of high amounts of practice in case of male pianists, β = − 1.008 [− 2.113, 0.134], Odds Ratio = 0.365 [0.121, 1.143]. However, this latter effect might occur with an estimated probability of 89.5%.

**Table 3:** Logistic regression models

	β Coefficients				Odds ratios		
	Estimate	SE	CI lower	CI upper	Estimate	CI lower	CI upper
Intercept	− 0.635	0.428	− 1.455	0.206	0.530	0.233	1.229
pract_q	1.017	0.454	0.127	1.896	2.765	1.135	6.656
male	0.598	0.548	− 0.472	1.658	1.818	0.624	5.247
start_age	0.152	0.270	− 0.368	0.688	1.164	0.692	1.989
pract_q:male	− 1.008	0.571	− 2.113	0.134	0.365	0.121	1.143

*N* = 67 consisting of 33 MD pianists and 34 pianists randomly selected from the HC group; s*tart_age* = age at which pianists started practicing music; *pract_q* = average amount of practice hours per day during timeframe 4 (i.e., 21–30 years of age). The model takes female pianists from the HC group as a reference. Predictors were standardized across participants. A bootstrap procedure was run, sampling data with replacement for 1000 iterations. Beta coefficients and odds ratios are reported together with 95 Confidence Intervals (CI)

## Discussion

Our study aimed at investigating the role of instrumental practice as a trigger factor of Musicians’ Dystonia. For this, we assessed differences in practice behavior between pianists affected by Musicians’ Dystonia (MD) and Healthy Controls (HC) in the years preceding the onset of the disease, namely between 5 and 30 years of age. To ensure comparability between the groups, we only included professional pianists in our study. Furthermore, we only included pianists in the MD group who had developed the disease after 30 years of age, for reasons described above.

We found that pianists with MD increased the amount of daily practice time to a greater extent between ages 5 and 30 than healthy pianists (see Fig. 1). However, subsequent analyses indicated that practice behaviors between both groups did not differ significantly in childhood and adolescence. Rather, pianists with MD had practiced significantly more than their healthy colleagues in the second and especially in the third decades of life.

Interestingly, we furthermore found that within the group of healthy pianists, only the males also significantly increased their practice time around the age of 20 years, while their female counterparts showed no such increase. Since several cases have been reported in which MD was preceded by a sudden, substantial increase in daily practice time, this has been widely considered a risk factor (Altenmüller and Jabusch 2010; Conti et al. 2008). Our observation may therefore suggest that the fourfold higher prevalence of MD in men as compared to women (Jabusch et al. 2005; Lim and Altenmüller 2003a) is at least partly due to a higher risk practice behavior in terms of a sudden increase in practice time in young adulthood. Until now, this has never been systematically investigated in MD (Rozanski et al. 2015). However, it would be in line with a study in patients with Writer’s Cramp (WC), a task-specific dystonia similar to MD, that found a significant increase in writing burden during the year before WC onset (Roze et al. 2009). Interestingly, in WC, it also has been noted that the prevalence among genders over the centuries depended on whether women or men held the greater proportion of writing professions, hinting again at the central role of repetitive movements’ burden in eliciting focal dystonia (Sheehy and Marsden 1982).

The altered practice behavior we found in male musicians compared to their female counterparts might also reflect a more competitive behavior of men, which has been found in sports sciences (Deaner 2013; Nicholls et al. 2007; Ogles and Masters 2003).

Accordingly, it is conceivable that psychological traits such as perfectionism and anxiety that have been identified as risk factors for MD (Altenmüller and Jabusch 2009) exert their effects mediated through their impact on practice behavior as has already been postulated (Sadnicka et al. 2018). This is also supported by the fact that that these effects were partly not reproducible or only had a low impact (Alpheis et al. 2022). In addition, the consideration that psychological factors indirectly influence the risk of MD via practice behavior is consistent with a study that has shown that anxiety in musicians may lead to more repetitive practice behavior (Passarotto et al. 2023).

That, in turn, highly repetitive movement execution over a prolonged period of time can lead to the development of focal dystonia by the primate model mentioned above (Byl et al. 1996). Clinically, it is also consistent with the notion that MD usually affects the body part that has the highest spatiotemporally critical load on fine motor skills, i.e., the left hand in violinists, the right hand in guitarists and pianists, and the embouchure in brass players (Altenmüller and Jabusch 2010; Rozanski et al. 2015). Furthermore, it can be observed that MD occurs preferentially in those instrument groups that have particularly high temporo-spatial demands (Rozanski et al. 2015), which in turn points to the crucial role of repetitive movement execution in the genesis of task-specific dystonias like MD (Sadnicka et al. 2018), which is consistent with our observations in this study.

According to these observations, it would be important to already design the practice times of aspiring musicians accordingly to minimize the risk of developing MD. This includes—in addition to varied practice strategies instead of highly repetitive approaches—avoiding too high an increase in daily practice time from young adulthood compared to adolescence. Music educators, who exert a high degree of influence on their students’ practicing, should also be educated about the consequences of aspiring professional musicians' practicing behavior over the course of their lives in relation to their risk for MD.

Unlike previous studies (i.e., Schmidt et al. 2013), we did not find a significantly higher age of onset of instrumental playing in patients with MD, even if there was a small trend in that direction. This might be due to our smaller sample size.

The study comes with several limitations: it is based on retrospective estimation of practice behaviors and its results might be affected by recall biases. Furthermore, the sample includes only 11 female participants affected by MD, making the gender-related statistical analyses less robust. Moreover, the study investigates practice behaviors only up to the age of 30 years, i.e., below the median onset age of MD (33–35) (Conti et al. 2008; Granert et al. 2011; Hirata et al. 2004; Lederman 1991; Tubiana 2003), rather than the exact age at which individual participants noticed the first motor symptoms and coordination problems. It is therefore not possible based on our study to evaluate whether intensive practice in a short-term interval leads to the onset of the disease, or whether high daily practice times in early adulthood promote the disease. Nevertheless, 51.5% of the MD patients who participated in the study developed the disease within 10 years after the period examined in the study, between 30 and 40 years of age. This is not conclusive proof of causality, but it surely supports the idea that practice behaviors might play a direct role in the pathogenesis of MD. Additionally, since we included only pianists in the MD group who developed the disease after the age of 30, it is possible that we thereby excluded a larger proportion of patients with a genetic predisposition and studied mainly those who developed MD less for genetic and more for behavioral reasons, since previous studies have shown that patients with a genetic predisposition develop MD earlier than those without a genetic predisposition. (Doll-Lee et al. 2023). Among the same line, the study did not include family or genetic testing to check for increased risk for the development of MD, which might also have played a role in disease development. In addition, other than the prerequisite professional level of the participants, no analyses were conducted on skill, environmental challenges (temperature of the room, size of the room, and protected sound space), psychological stressors, or quality of the biomechanics during practice time, all of which may have exacerbated the negative effects of the increased practice time.

## Conclusion

Our study shows that practice behavior could be a risk factor for the occurrence of MD and that men seem to show a more risky practice behavior in this sense, which may be a possible explanation of the higher prevalence of MD in men. To the authors’ knowledge, this represents the first empirical evidence of the role of dysfunctional practice behaviors in triggering MD. The identification of behavioral risk factors is of relevance, because no causal therapy options in MD exist and, therefore, our focus should lie on primary prevention measures. Considering previous findings as well as our results, these should include education on diversified practice strategies from the earliest stages of instrumental instruction to avoid the accumulation of repetitive movement exertion. Moreover, music pedagogues should be made aware that highly repetitive practice patterns can have a devastating effect on musicians' later careers and health.

## Figures and Tables

**Fig. 2:**
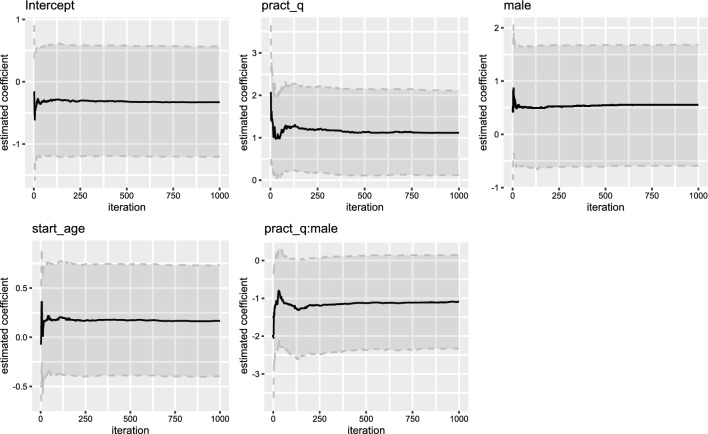
Estimated regression coefficients at different number of sampling iterations. s*tart_age* = age at which pianists started practicing music; *pract_q* = average amount of practice hours per day during the 21–30 years of age timeframe

## Data Availability

The data that support the findings of this study are available from the corresponding author upon reasonable request.

## Appendix

See Tables

**Table 4:** Group differences in daily practice quantity at different timeframes (*tf*)

	Estimate	Est. Error	CI lower	CI upper
Fixed effects				
tf1	1.03	0.13	0.78	1.3
tf2	2.10	0.22	1.67	2.56
tf3	3.47	0.33	2.83	4.11
tf4	3.57	0.29	3.00	4.14
tf1:md_group	0.12	0.25	− 0.36	0.59
tf2:md_group	0.29	0.34	− 0.37	0.97
tf3:md_group	0.51	0.54	− 0.56	1.57
tf4:md_group	1.27	0.53	0.22	2.34
tf1:males	− 0.19	0.19	− 0.56	0.19
tf2:males	0.26	0.27	− 0.27	0.80
tf3:males	0.28	0.44	− 0.58	1.15
tf4:males	1.11	0.42	0.28	1.95
tf1:md_group:males	− 0.26	0.32	− 0.88	0.37
tf2:md_group:males	− 0.83	0.45	− 1.70	0.04
tf3:md_group:males	− 0.62	0.71	− 2.00	0.76
tf4:md_group:males	− 1.06	0.69	− 2.45	0.30

Performer	Estimate	Est.Error	l− 95% CI	u− 95% CI
Random effects				
sd(tf1)	0.52	0.08	0.37	0.68
sd(tf2)	1.08	0.10	0.90	1.29
sd(tf3)	1.63	0.14	1.38	1.92
sd(tf4)	1.43	0.12	1.20	1.70
cor(tf1, tf2)	0.80	0.10	0.58	0.96
cor(tf1, tf3)	0.56	0.12	0.30	0.77
cor(tf1, tf4)	0.73	0.07	0.58	0.84
cor(tf2, tf3)	0.34	0.14	0.06	0.60
cor(tf2, tf4)	0.22	0.12	− 0.02	0.44
cor(tf3, tf4)	0.51	0.09	0.31	0.67
Sigma	0.40	0.03	0.35	0.46
Coefficients of determination				
Conditional R2	0.95	0.01	0.94	0.97
Marginal R2	0.52	0.03	0.48	0.57

*N* = 82

The model takes female pianists from the HC group as a reference. Individual practice behaviors can be estimated by summing the corresponding fixed effects coefficients. For instance, the amount of daily practice achieved by a female pianist from MD group during *timeframe3* can be estimated through the following formula, tf3 + tf3:md_group = 3.47 + 0.51 = 3.98. For further information, see Sects. 2.3 Materials and 2.5 Data analyses

*tf* timeframe; *CI* credible interval

4 and

**Table 5:** Descriptive statistics for daily practice quantity of female and male participants in the MD and HC groups during each timeframe

	MD group (*N* = 33)		HC group (*N* = 49)	
	Females (*N* = 11)	Male (*N* = 22)	Female (*N* = 26)	Male (*N* = 23)
Timeframe 1	1.10 (0.61)	0.70 (0.34)	1.07 (0.83)	0.81 (0.65)
Timeframe 2	2.50 (1.10)	1.50 (0.82)	2.10 (1.41)	2.17 (1.13)
Timeframe 3	4.09 (1.30)	3.32 (1.84)	3.47 (1.86)	3.57 (1.43)
Timeframe 4	4.95 (1.04)	4.57 (1.69)	3.57 (1.63)	4.50 (1.14)

*N* = 82; the table reports mean and standard deviation (in brackets) of *daily practice quantity* values for each subgroup

5; Fig. 2.
